# Unbiased K-L estimator for the linear regression model

**DOI:** 10.12688/f1000research.54990.1

**Published:** 2021-08-19

**Authors:** BENEDICTA Aladeitan, Adewale F Lukman, Esther Davids, Ebele H Oranye, Golam B M Kibria

**Affiliations:** 1 Physical Sciences, Landmark University, Omu-Aran., Kwara State, +234, Nigeria; 2 Biostatistics and Epidemiology, University of Medical Sciences., Ondo, Ondo State, +234, Nigeria; 3 Statistics, University of Nigeria, Nsukka, Enugu State, +234, Nigeria; 4 Mathematics and Statistics, Florida International University, Florida, USA, USA

**Keywords:** Linear regression model, Ordinary Least Square estimator, Ridge regression, K-L estimator, High Heating values, Proximate analysis.

## Abstract

**Background: **In the linear regression model, the ordinary least square (OLS) estimator performance drops when multicollinearity is present. According to the Gauss-Markov theorem, the estimator remains unbiased when there is multicollinearity, but the variance of its regression estimates become inflated. Estimators such as the ridge regression estimator and the K-L estimators were adopted as substitutes to the OLS estimator to overcome the problem of multicollinearity in the linear regression model. However, the estimators are biased, though they possess a smaller mean squared error when compared to the OLS estimator.

**Methods:** In this study, we developed a new unbiased estimator using the K-L estimator and compared its performance with some existing estimators theoretically, simulation wise and by adopting real-life data.

**Results:** Theoretically, the estimator even though unbiased also possesses a minimum variance when compared with other estimators.  Results from simulation and real-life study showed that the new estimator produced smaller mean square error (MSE) and had the smallest mean square prediction error (MSPE). This further strengthened the findings of the theoretical comparison using both the MSE and the MSPE as criterion.

**Conclusions: **By simulation and using a real-life application that focuses on modelling, the high heating values of proximate analysis was conducted to support the theoretical findings. This new method of estimation is recommended for parameter estimation with and without multicollinearity in a linear regression model.

## Introduction

Considering the general linear regression model


$$y=X\beta +\varepsilon_{i}\;(1)$$

such that *ε _i_* is normally distributed with mean *0* and variance *σ* ^2^ *I* where *I* is the identity matrix. *y* is an *n* × *1* vector of dependent variable, *X* is an n × *p* matrix of the independent variables, *β* is a *p* × *1* vector of unknown regression parameters of interest. The method of ordinary least square (OLS) is well known and generally accepted for estimating the parameters ( *β*’s) in the linear regression model. The model is defined as:


$${\hat{\beta }}_{OLS}=H^{-1}X^{\prime }y\;(2)$$

Where *H* = $X^{\prime }X$ and ${\hat{\beta }}_{OLS}$ is normally distributed that is ${\hat{\beta }}_{OLS}$ ~ *N*( *β*, *σ* ^2^ *H* ^–1^). However, when the OLS estimator is applied to a model where there is correlation between the independent variables, then the variance of the regression estimates becomes inflated ^1, 2^. This relationship between the independent variables is referred to as multicollinearity ^3, 4^.

In addressing the problem of multicollinearity, various biased estimators with mean square error smaller than the OLS have been developed by different authors ^2– 15^. The limitations of these estimators is that they are biased , however the unbiased versions of some of them have been developed. The advantage of these estimators is that they produced estimates that were similar to the OLS estimator with better mean squared error. Crouse *et al.* ^16, 17^ developed the unbiased ridge and the Liu estimators. Wu ^18^ developed the unbiased version of the two-parameter estimator by Ozkale and Kaciranlar ^9^. Lukman *et al.* ^19^ developed the unbiased modified ridge-type estimator. Recently, the K-L estimator was proposed to circumvent the problem of multicollinearity in the linear regression model ^13^. The K-L estimator is classified as a biased estimator with a single biasing parameter ^13^. 

In this study, a new unbiased technique is developed based on the K-L estimator and its properties are derived. We compared the unbiased K-L estimator with some existing techniques using the mean square error (MSE) criterion.

## Methods

### Unbiased K-L estimator with prior information

Hoerl and Kennard ^5^ developed the ridge estimator to mitigate multicollinearity in the linear regression model. The ridge estimator of *β* with the biasing parameter *k* is:


$${\hat{\beta }}_{RR}(k)={\left(H+kI\right)}^{-1}X^{\prime }y,\;k>0\;(3)$$

The modified ridge technique was proposed with the addition of the prior information ^6^. This is expressed as follows: 


$${\hat{\beta }}_{MRE}(k,b)={\left(H+kI\right)}^{-1}(X^{\prime }y+kb)\;(4)$$

According to 16, the unbiased ridge estimator with the introduction of prior information *J* is given as


$${\hat{\beta }}_{UMRE}={\left(H+kI\right)}^{-1}(X^{\prime }y+kJ)\;(5)$$

where *J* and ${\hat{\beta }}_{OLS}$ are uncorrelated and *J* ~ N( *β*, *D*) such that $D=(\frac{\sigma^{2}}{k})I_{p}$ and *I _p_* is *p* × *p* identity matrix. *J* is estimated by $J=\frac{\sum_{i=1}^{p}{\hat{\beta }}_{i}}{p}$ .

Modified ridge-type method proposed by 3 is given as follows:


$${\hat{\beta }}_{MRT}(k,d)={\left[H+k(1+d)\right]}^{-1}S{\hat{\beta }}_{OLS}=A_{kd}{\hat{\beta }}_{OLS}\;(6)$$

where *A _kd_* = [ *S*+ *k*(1+ *d*)] ^–1^S.

The unbiased modified ridge type estimator ^19^ was developed and defined as follows:


$${\hat{\beta }}_{UMRT}(A_{kd},J)=A_{kd}{\hat{\beta }}_{OLS}+(I-A_{kd})J={\hat{\beta }}_{MRT}(k,d)+(I-A_{kd})J\;(7)$$

where *A _kd_* = [ *H*+ *k*(1+ *d*)] ^–1^ *H* such that $D=\frac{\sigma^{2}}{k(1+d)}$ . Consequently, $J\sim N\left(\beta ,\frac{\sigma^{2}}{k(1+d)}\right)$ for

*k* ˃ 0, 0< *d*<1.

Recently ^13^, proposed the K-L estimator and found that this estimator generally outperform the ridge regression estimator. The K-L estimator of *β* is defined as:


$${\hat{\beta }}_{KL}(k)={\left(H+kI\right)}^{-1}(H-kI){\hat{\beta }}_{OLS}=A_{k}{\hat{\beta }}_{OLS}\;k>0\;(8)$$

where *A _k_* = ( *H* + *kI*) ^-1^ ( *H* – *kI*)

This research proposes an unbiased K-L estimator following the convex method. The convex method is defined as: 


$$\hat{\beta }(G,J)=G{\hat{\beta }}_{OLS}+(I-G)J\;(9)$$

where *G* is a *p*× *p* matrix and *I* is an identity matrix of *p*× *p* dimensions. Thus, the MSE of $\hat{\beta }$ ( *G*, *J*) is


$$MSE(\hat{\beta }(G,J))=\sigma^{2}GH^{-1}G^{\prime }+(I-G)D(I-G{)}^{\prime }\;(10)$$

Such that,


$$\frac{\partial_{MSE}(\beta (G,J))}{\partial G}=2G(\sigma^{2}H^{-1}+D)-2D=0\;(11)$$

The value of *G* from ( 11) is *G* = *D*( *σ* ^2^ *H* ^–1^ + *D*) ^–1^. Accordingly, *D* = *σ* ^2^( *I* – *G*) ^-1^ *GH* ^-1^). We observed that the convex estimator *β*( *G*, *J*) is an unbiased estimator of *β* and possesses minimum MSE for optimal value of *G*. Consequently, the new unbiased estimator is defined as


$${\hat{\beta }}_{UKL}(k,J)=A_{k}{\hat{\beta }}_{OLS}+(I-A_{k})J={\hat{\beta }}_{KL}(k)+(I-A_{k})J\;(12)$$

where *A _k_* = ( *H* + *kI*) ^–1^( *H* – *kI*) and the value of $V=\frac{\sigma^{2}(S-kI)S^{-1}}{2k}$ . Therefore, $J\sim N\left(\beta ,\frac{\sigma^{2}(S-kI)S^{-1}}{2k}\right)$ for *k* ˃ 0.

It can be expressed conveniently that ${\hat{\beta }}_{UKL}(k,J)$ is unbiased for *β* The new estimator has properties defined as follows:


$$\begin{array}{l} \;E({\hat{\beta }}_{UKL}(k,J))=E({\hat{\beta }}_{KL}(k)+(I-A_{k})J)\\ =E(A_{k}{\hat{\beta }}_{OLS}+[I-A_{k}]J)\;\text{where}\;E({\hat{\beta }}_{OLS})=E(J)=\beta \\ =A_{k}\beta +(I-A_{k})\beta ]\\ =\beta \end{array}\;(13)$$

It follows from Equation (13) that the proposed estimator is unbiased. This classified the new estimator into the same class with the OLS estimator. The biasedness of the estimator is also zero. This is proved as follows:


$$Bias({\hat{\beta }}_{UKL}(k,J))=E({\hat{\beta }}_{UKL}(k,J))-\beta =\beta -\beta =0\;(14)$$

$$MSE(\beta_{UKL}(k,J))=D(\beta_{KL}(k)+(I-A_{k})J\text{)}=\frac{\sigma^{2}(H-kI)}{H(H+kI)}\;(15)$$

Given that there exists an orthogonal matrix *Q,* such that $Q^{\prime }X^{\prime }XQ$ = *Ε* = *diag* ( *e*_1_, *e*_2_,..., *e _p_*) where *e _i_* is the *i* ^th^ eigenvalue of $X^{\prime }X$ , *E* and *Q* are the matrices of eigenvalues and eigenvectors of $X^{\prime }X$ respectively. Equation (1) can be expressed canonically as:


y=Zα+ε(16)

where *Z* = *XQ*, $\alpha =Q^{\prime }\beta$ and $Z^{\prime }Z=E$ = *Ε*. For Equation (15), we get the following representations:


$${\hat{\alpha }}_{OLS}=E^{-1}Z^{\prime }y\;(17)$$

$${\hat{\alpha }}_{RR}(k)={\left(E+kI\right)}^{-1}Z^{\prime }y\;(18)$$

$${\hat{\alpha }}_{UMRE}(k)={\left[E+kI\right]}^{-1}(Z^{\prime }y+kJ)\;(19)$$

$${\hat{\alpha }}_{KL}(k)={\left(E+kI\right)}^{-1}(E-kI)Z^{\prime }y\;(20)$$

$${\hat{\alpha }}_{UKL}(k,J)={\hat{\alpha }}_{KL}(k)+(I-A_{k})J\;(21)$$

**Lemma 1.1** Let N be an *n*× *n* positive definite matrix and *α* be some vector, then $N-\alpha \alpha^{\prime }\geq 0$ if and only if $\alpha^{\prime }N^{-1}\alpha \leq 1$ ^20^.

**Lemma 1.2** Let ${\hat{\alpha }}_{i}$ = *C _i_y i* = 1, 2 be two linear estimators of *α*. Suppose that *D* = *Cov*( ${\hat{\alpha }}_{1}$ ) – *Cov*( ${\hat{\alpha }}_{2}$ ) > 0, where *Cov*( ${\hat{\alpha }}_{i}$ ), *i* = 1, 2 denotes the covariance matrix of ${\hat{\alpha }}_{i}$ and *bias*( ${\hat{\alpha }}_{i}$ ) = b = ( *C _i_X* – *I*) *α*, *i* = 1, 2. Consequently,


$$({\hat{\alpha }}_{1}-{\hat{\alpha }}_{2})=MSEM({\hat{\alpha }}_{1})-MSEM({\hat{\alpha }}_{2})=\sigma^{2}D+b_{1}b_{1}^{\prime }-b_{2}b_{2}^{\prime }>0\;(22)$$

if and only if $b_{2}^{\prime }{\left[\sigma^{2}D+b_{1}b_{1}^{\prime }\right]}^{-1}b_{2}<1$ where $MSEM({\hat{\alpha }}_{i})=Cov({\hat{\alpha }}_{i})+b_{i}b_{i}^{\prime }$ ^21^.

### Theoretical comparison

${\hat{\alpha }}_{\mathrm{OLS}}$ ***and*** ${\hat{\alpha }}_{\mathrm{UKL}}(k,J)$

*Theorem 1.1*. ${\hat{\alpha }}_{UKL}(k,J)$ is preferred to ${\hat{\alpha }}_{OLS}$ by using the matrix mean square error as criteria for *k* > *0*. 

*Proof*

Recall that,


$$MSEM({\hat{\alpha }}_{OLS})=\sigma^{2}E^{-1}\;(23)$$

$$MSEM{\hat{\alpha }}_{UKL}(k,J)=\sigma^{2}{\left(E+kI\right)}^{-1}(E-kI)E^{-1}\;(24)$$

The difference between ( 23) and ( 24) is as follows:


$$\begin{array}{l} MSEM({\hat{\alpha }}_{OLS})-MSEM({\hat{\alpha }}_{UKL}(k,J))=\sigma^{2}E^{-1}-\sigma^{2}{\left(E+k\right)}^{-1}(E-k)E^{-1}\\ \;=\sigma^{2}[E^{-1}-{\left(E+k\right)}^{-1}(E-k)E^{-1}]\\ \;=\sigma^{2}diag\left[\frac{1}{e_{i}}-\frac{\left(e_{i}-k\right)}{e_{i}(e_{i}+k)}\right] \end{array}\;(25)$$

Simplifying ( 25) further, we observed that *E* ^–1^ – ( *E* + *k*) ^–1^ ( *E* – *k*)Λ ^–1^ will be positive definite since 2 *k* > 0 for *k* > *0.*

${\hat{\alpha }}_{RR}(k)$ ***and*** ${\hat{\alpha }}_{\mathrm{UKL}}(k,J)$

*Theorem 3.2.* ${\hat{\alpha }}_{UKL}(k,J)$ is preferred to ${\hat{\alpha }}_{RR}(k)$ by using the matrix mean square error as criteria for *k* > *0*. 

*Proof*


$$MSEM({\hat{\alpha }}_{RR}(k))=\sigma^{2}B_{k}EB_{k}^{\prime }+k^{2}B_{k}\alpha \alpha^{\prime }B_{k}^{\prime }\;(26)$$

where *B _k_* = ( *E* + *kI*) ^–1^

The difference of Equation (24) and ( 26) is as follows:


$$\begin{array}{l} MSEM({\hat{\alpha }}_{RR}(k))-MSEM({\hat{\alpha }}_{UKL}(k,J))=\sigma^{2}E{\left(E+k\right)}^{-2}-\sigma^{2}{\left(E+k\right)}^{-1}(E-k)E^{-1}\\ \;=\sigma^{2}[E{\left(E+k\right)}^{-2}-{\left(E+k\right)}^{-1}(E-k)E^{-1}]\\ \;=\sigma^{2}diag\left[\frac{e_{i}}{{\left(e_{i}+k\right)}^{2}}-\frac{\left(e_{i}-k\right)}{e_{i}(e_{i}+k)}\right]\; \end{array}\;(27)$$

Simplifying ( 27) further, we observed that *E*( *E* + *k*) ^–2^ – ( *E* + *k*) ^–1^ ( *E* – *k*) *E* ^–1^ will be positive definite since *k* ^2^ > 0.

${\hat{\alpha }}_{\mathrm{URR}}(k)$ **and** ${\hat{\alpha }}_{\mathrm{UKL}}(k,J)$

*Theorem 3.3.* ${\hat{\alpha }}_{UKL}(k,J)$ is preferred to ${\hat{\alpha }}_{URR}(k)$ by using the matrix mean square error as criteria for *k* > *0*. 

*Proof*


$$MSEM\;({\hat{\alpha }}_{URR}(k))=\sigma^{2}{\left(E+kI\right)}^{-1}\;(28)$$

$$\begin{array}{l} MSEM\;({\hat{\alpha }}_{URR}(k))-MSEM({\hat{\alpha }}_{UKL}(k,J))=\sigma^{2}{\left(E+k\right)}^{-1}-\sigma^{2}{\left(E+K\right)}^{-1}(E-k)E^{-1}\\ \;=\sigma^{2}diag\left[\frac{1}{\left(e_{i}+k\right)}-\frac{\left(e_{i}-k\right)}{e_{i}(e_{i}+k)}\right] \end{array}\;(29)$$

We observed that *σ* ^2^( *E* + *k*) ^–1^ – *σ* ^2^( *E* + *k*) ^–1^( *E* – *k*) *E* ^–1^ will be positive definite since *k* > *0.*

${\hat{\alpha }}_{\mathrm{KL}}(k)$ **and** ${\hat{\alpha }}_{\mathrm{UKL}}(k,J)$

*Theorem 3.4*. ${\hat{\alpha }}_{UKL}(k,J)$ is preferred to ${\hat{\alpha }}_{KL}(k)$ by using the matrix mean square error as criteria for *k* > *0*. 

*Proof*


$$MSEM\;{\hat{\alpha }}_{KL}(k)=\sigma^{2}E_{k}\left(E-k\right)\Lambda^{-1}E_{k}\left(E-k\right)+4k^{2}E_{k}\alpha \alpha^{\prime }E_{k}\;(30)$$

where *E _k_* = ( *E* + *k*) ^–1^. Consequently,


$$\begin{array}{l} MSEM\;({\hat{\alpha }}_{KL}(k))-MSEM({\hat{\alpha }}_{UKL}(k,J))=\sigma^{2}{\left(E-k\right)}^{2}E^{-1}{\left(E+k\right)}^{-2}-\sigma^{2}(E-k)E^{-1}{\left(E+k\right)}^{-1}\\ \;=\sigma^{2}diag\left[\frac{{\left(e_{i}-k\right)}^{2}}{e_{i}{\left(e_{i}+k\right)}^{2}}-\frac{\left(e_{i}-k\right)}{e_{i}(e_{i}+k)}\right] \end{array}\;(31)$$

We observed that *σ* ^2^( *E* – *k*) ^2^ *E* ^–1^( *E* + *k*) ^–2^ – *σ* ^2^( *E* – *k*) *E* ^–1^( *E* + *k*) ^–1^ will be positive definite if *k* > 2Λ for *k* > *0*.

### Selection of the biasing parameters

In this study, we adopt the following biasing parameter for the ridge and the unbiased ridge estimators:


$$\hat{k}=\frac{p{\hat{\sigma }}^{2}}{\sum_{i=1}^{p}{\hat{\alpha }}_{i,OLS}^{2}}\;(32)$$

For the K-L estimator, we adopted:


$${\hat{k}}_{r}=min\left[\frac{{\hat{\sigma }}^{2}}{2{\hat{\alpha }}_{i,OLS}^{2}+\frac{{\hat{\sigma }}^{2}}{e_{i}}}\right]\;(33)$$

For the proposed estimator, the following biasing parameters were examined:


$$\text{UKL-1};{\hat{k}}_{r}=min\left[\frac{{\hat{\sigma }}^{2}}{2{\hat{\alpha }}_{i,OLS}^{2}+\frac{{\hat{\sigma }}^{2}}{e_{i}}}\right]\;(34)$$

$$\text{UKL-2};{\hat{k}}_{1}=max\left[\frac{1}{{\hat{\alpha }}_{i,OLS}^{2}}\right]\;(35)$$

$$\text{UKL-3};{\hat{k}}_{2}=\sqrt{max\left(\frac{{\hat{\sigma }}^{2}}{{\hat{\alpha }}_{i,OLS}^{2}}\right)}\;(36)$$

$$\text{UKL-4};k=\frac{p\sigma^{2}}{\sum_{i=1}^{p}{\hat{\alpha }}_{i,OLS}^{2}}\;(37)$$

## Results

R Studio was used for both the simulation and real-life analysis. The independent variables were generated by following the study of McDonald and Galarneau ^22^ given as:


$$X_{ij}={\left(1-r^{2}\right)}^{1/2}z_{ij}+rz_{i(p+1)}\;(38)$$

where *Z _ij_* are independent standard normal pseudorandom numbers, *r* ^2^ is the relationship between any two independent variables and *p* is the number of independent variables taken as three and seven in this study. The values of *r* ^2^ varies between 0.8, 0.9, 0.99 and 0.999 respectively. For *p*=3, the response variable is defined as:


$$Y_{1}=\beta_{1}X_{1}+\beta_{2}X_{2}+\beta_{3}X_{3}+e_{i}\;(39)$$

where *e _i_* is normally distributed with mean 0 and variance *σ* ^2^. *β* is chosen such that $\beta^{\prime }\beta$ = 1 ^23^. Samples of size 30, 50, and 100 were used. Values of *σ* are 1 and 5. The mean square error is calculated as:


$$MSE(\hat{\alpha })=\frac{1}{2000}\sum_{j=1}^{2000}\left({\hat{\beta }}_{ij}-\beta_{i}{)}^{\prime }({\hat{\beta }}_{ij}-\beta_{i}\right)\;(40)$$

where ${\hat{\beta }}_{ij}$ is the estimate of the i ^th^ parameter in j ^th^ replication and *β _i_* are the true parameter values. The MSE results are presented in Table 1 and Table 2. We observed the following:

**Table 1.** Estimated MSE for the simulation study when sigma=1.

*P*	n	ρ	OLS	Ridge	U-Ridge	KL	UKL1	UKL2	UKL3	UKL4
3	30	0.8	1.2575	1.1783	1.2011	1.2086	1.2235	1.1140	1.0996	1.1589
		0.9	3.7789	3.4543	3.5538	3.5654	3.6322	2.2056	2.9748	3.3636
		0.99	5.6205	3.2848	3.8963	3.9439	4.4144	1.9226	2.0469	2.8841
		0.999	35.7111	12.5735	18.3843	19.5479	24.0513	4.6898	4.5471	8.6644
	50	0.8	3.1817	2.9869	3.0504	3.0704	3.1070	1.8099	2.6533	2.9248
		0.9	3.1387	2.8611	2.9496	2.9555	3.0146	1.8909	2.5800	2.7765
		0.99	4.7483	3.2525	3.6586	3.6869	3.9912	2.0781	2.4117	2.9391
		0.999	21.4035	8.1820	11.4916	12.2398	14.7889	3.5507	3.4045	5.9699
	100	0.8	4.0550	3.9420	3.9793	3.9983	4.0171	2.5400	3.5609	3.9050
		0.9	1.9893	1.8763	1.9128	1.9249	1.9460	1.2788	1.6186	1.8409
		0.99	5.2450	4.1823	4.4893	4.4379	4.6780	2.9884	3.4428	3.9226
		0.999	14.0023	5.2709	7.4834	7.8224	9.5428	1.7324	1.6713	3.7563
7	30	0.8	4.0218	3.0750	3.1889	3.5199	3.5851	1.3614	1.9079	2.6116
		0.9	4.7904	3.2139	3.3907	3.9152	4.0246	1.7000	1.9975	2.5728
		0.99	19.0010	6.7494	7.9171	12.1457	12.9583	8.1601	7.1691	3.8239
		0.999	159.6416	41.2828	52.2229	93.3516	101.1318	76.3306	73.9092	16.4346
	50	0.8	3.5577	2.8785	2.9656	3.2243	3.2697	1.5240	1.7956	2.4915
		0.9	5.4123	4.0309	4.2014	4.6759	4.7732	1.4829	2.1778	3.3137
		0.99	13.4754	4.6632	5.5231	8.6238	9.2025	6.0085	4.6971	2.3589
		0.999	121.0170	33.8018	42.0867	73.0005	78.7071	55.0748	53.4195	13.2499
	100	0.8	3.8136	3.4409	3.4917	3.6804	3.6991	1.1615	2.3239	3.1992
		0.9	4.0491	3.4280	3.5095	3.7816	3.8185	1.3207	2.2128	3.0562
		0.99	7.5441	3.9596	4.3415	5.4878	5.7412	2.9686	2.6924	2.7004
		0.999	42.4513	12.1138	15.0220	24.9592	27.0472	18.0973	16.6656	4.7009

**Table 2.** Estimated MSE for the simulation study when sigma=5.

*p*	N	ρ	OLS	Ridge	U-Ridge	KL	UKL1	UKL2	UKL3	UKL4
3	30	0.8	6.2685	2.9512	3.8065	3.934	4.5876	3.4963	2.1897	2.3464
		0.9	12.7983	6.1772	7.9023	8.2484	9.5299	6.7402	3.9396	4.9341
		0.99	86.9643	28.7447	43.3680	46.5598	57.8349	8.9211	6.2380	18.9046
		0.999	837.2886	261.9639	405.9761	437.9803	549.2998	62.0722	86.7562	165.7144
	50	0.8	5.8175	3.4525	4.1075	4.2205	4.6864	3.7063	3.1453	2.9308
		0.9	7.7474	4.0443	5.0257	5.2374	5.9512	4.2569	3.0837	3.3158
		0.99	48.2308	16.4888	24.4289	26.4166	32.4916	6.0867	4.03304	11.1893
		0.999	464.4477	143.1775	223.0953	243.7469	305.1109	35.2421	47.7284	90.4319
	100	0.8	5.5264	3.8849	4.3618	4.3957	4.7344	4.2461	3.9482	3.4782
		0.9	5.0219	2.6888	3.3142	3.3498	3.8251	3.4881	2.5229	2.2156
		0.99	33.7948	12.2441	17.6493	18.6328	22.8495	9.0504	3.5528	8.6170
		0.999	304.1409	89.4080	142.8193	153.6682	195.4571	12.0858	27.2452	54.1628
7	30	0.8	24.8444	8.5389	10.1199	15.8535	16.9275	7.0383	5.6513	4.3758
		0.9	44.3043	13.6507	16.5797	27.3928	29.4076	11.5369	10.8441	6.2566
		0.99	396.6272	105.4977	132.8158	235.8310	254.8663	125.2039	167.0124	40.2664
		0.999	3912.3412	1018.8512	1289.3604	2316.7446	2505.2394	1816.5141	1944.9425	380.5377
	50	0.8	17.5698	6.4007	7.5074	11.4719	12.2056	5.4997	3.8656	3.3115
		0.9	33.4275	11.1706	13.3419	21.3280	22.7780	9.3004	7.1973	5.3547
		0.99	293.0569	78.6266	98.9722	175.8942	189.8296	67.7918	110.9219	28.3353
		0.999	2915.6689	775.1140	978.3931	1745.1447	1884.4693	1239.4901	1424.9954	271.2904
	100	0.8	8.9550	4.4238	4.9084	6.4437	6.7548	3.4211	2.8158	2.8143
		0.9	13.9441	5.7784	6.6171	9.4022	9.9580	4.9346	3.3490	3.2245
		0.99	102.2963	29.2440	36.3967	61.5713	66.4953	19.4200	26.6929	9.8965
		0.999	981.0459	263.5057	333.4068	580.2188	628.6188	322.2568	448.7248	77.0239

1. All other alternative techniques studied in this work outperforms the OLS estimator at all the levels of multicollinearity.
2. The ridge estimator outperforms its unbiased version when the MSE is used as a criterion.
3. The proposed unbiased estimator (UKL) in this study outperform its K-L estimator counterpart.
4. There was a general better performance of the proposed estimator over all the estimators considered in this work though its performance is a function of the choice of biasing parameters.

In this study, the following trends about the mean square error and the factors in the simulation were observed:

1. There is a decrease in the MSE when there is an increase in the sample size at a particular level of multicollinearity.
2. Increase in the value of σ leads to a corresponding increase in the mean square errors of each of the estimators when other variables are kept constant.
3. An increase in the number of explanatory variables leads to a corresponding increase in the MSE of all estimators at varying level of multicollinearity and σ.

### Application: poultry waste data

The poultry waste data adopted in this study was found and analyzed in Qian *et al.* ^24, 25^ and was also recently employed by Lukman *et al.* ^19^. The study was aimed at modelling the high heating values of proximate-based model. The response variable is High Heating Values (HHV), while the independent variables are Fixed Carbon (FC), Volatile Matter (VM), and Ash (A). The linear regression model is:


$$HHV=\beta_{0}+\beta_{1}FC+\beta_{2}VM+\beta_{3}A+\varepsilon \;(41)$$

where *ε* is the normally distributed random error term. In this study, the Jarque-Bera (JB) test was employed to know the distribution of the residual. The test statistic and its p-value are 0.6409 and 0.7258, respectively. The result shows that the residual in the model is normally distributed. We diagnosed if the model has the problem of multicollinearity. According to Lukman *et al.* ^14^, the model suffered from the problem of multicollinearity because the variance inflation factors ( *VIF _FC_* =997.819, *VIF _VM_* =2163.504, *VIF _ASH_* =1533.782) are greater than ten (10). Also, there is evidence of multicollinearity with the use of the condition number (CN).

Following Lukman *et al.* ^3, 4^, moderate level of multicollinearity is observed if the CN ranges are between 100 and 1000 but severe multicollinearity is encountered if CN is greater than 1000. For effective modelling, we considered some alternative estimators to the ordinary least squared estimator in this study. These include the ridge estimator, unbiased ridge estimator, K-L estimator and the unbiased K-L estimator. The estimators’ performance was examined using the mean square error. We also adopt the leave-one-out cross-validation to validate how well the estimators perform ^14^. The performance of the estimator is assessed through the mean squared prediction error (MSPE). The estimator with the least MSE and MSPE is considered as the best. The result is available in Table 3.

**Table 3.** Regression coefficients and MSE.

coef.	${\hat{\alpha }}_{OLS}$	${\hat{\alpha }}_{RR}$	${\hat{\alpha }}_{URR}$	${\hat{\alpha }}_{KL}$	${\hat{\alpha }}_{UKL-1}$	${\hat{\alpha }}_{UKL-2}$	${\hat{\alpha }}_{UKL-3}$	${\hat{\alpha }}_{UKL-4}$
*α*_0_	167.72	102.099	167.72	144.50	167.72	167.72	167.72	167.72
*α*_1_	-1.2704	-0.6143	-1.2704	-1.038	-1.2704	-1.2704	-1.2704	-1.2704
*α*_2_	-1.5311	-0.8763	-1.5311	-1.299	-1.5311	-1.5311	-1.5311	-1.5311
*α*_3_	-1.6840	-1.0267	-1.6840	-1.451	-1.6840	-1.6840	-1.6840	-1.6840
MSE	4521.1	1675.79	2752.17	3925.5	3757.45	3269.29	983.24	1645.56
MSPE	349.92	154.97	210.41	78.33	282.07	109.08	5.891	17.706

From Table 3, the regression estimates of the following methods are the same: URR, UKL, and OLS as expected. They also possess a smaller mean squared error when compared with the OLSE. The estimators all exhibit the same regression coefficient signs. The proposed estimator UKL demonstrated the best performance in terms of the MSE and the MSPE. Although its performance is a function of the biasing parameter *k*. 

## Conclusion

There is high inconsistency in the performance of the OLS estimator for parameter estimation in the linear regression model with multicollinearity problem. The estimator is unbiased but no longer has minimum variance. Due to this setback, in this study, the unbiased K-L estimator was developed and the properties of this new estimator was derived and established. It was found that the estimator is in the class of the unbiased estimator. An added advantage of this estimator over the OLS estimator is that it possesses minimum variance when multicollinearity is present. The superiority of the proposed estimator over the existing methods was theoretically established. The estimator is preferred to other estimators considered in this study.

Furthermore, the simulation and real-life results strengthened the findings of the theoretical comparison in terms of the mean squared error and the mean square prediction error. We recommend this new estimator for parameter estimation in a linear regression model with and without multicollinearity. In further studies, we will extend the new unbiased estimator to other generalized linear models such as the logistic regression model, Beta regression model, Gamma regression model etc.

## Data availability

### Underlying data

Zenodo: Regression Model to Predict the Higher Heating Value of Poultry Waste from Proximate Analysis. http://doi.org/10.5281/zenodo.5078977 ^25^.

This project contains the following underlying data:

- hhv data.txt

Data are available under the terms of the Creative Commons Attribution 4.0 International license (CC-BY 4.0).
