# Supplementary material for: Unbiased K-L estimator for the linear regression model
Source: F1000Res. 2021 Aug 19;10:832. [Version 1] doi: 10.12688/f1000research.54990.1 (PMC8825663; doi:10.12688/f1000research.54990.1)
Supplement: Peer review history

# Peer review: Reviewer response for version 1

**DOI:** 10.5256/f1000research.58523.r92410

**Published:** 2021-09-16

**Authors:** Mohammad Arashi

**Affiliations:** 1 Department of Statistics, Faculty of Mathematical Sciences, Ferdowsi University of Mashhad, Mashhad, Iran

Report on the paper “Unbiased K-L estimator for the linear regression model”

 In this study, an unbiased estimator is developed for multicollinear situations. Analytical comparisons are carried out to demonstrate the superiority of the proposed estimator over some existing ones in the literature using the MSE of estimation and MSE of prediction. Numerical analyses, including Monte Carlo simulation and read data, are conducted to support the findings. The mathematical results sound correct, and the topic is eye-catching. Advancing estimation strategies to combat multicollinearity is improving; however, they are mostly biased. In this study, an unbiased version is proposed. I recommend making a minor revision addressing the following comments before acceptance.

1. Everywhere applies, change “ordinary least square” to “ordinary least square **s**”.
2. What is K-L in the Abstract? Whenever you use an abbreviation for the first time, define it completely.
3. The authors claim, “However, the estimators are biased, though they possess a smaller mean squared error when compared to the OLS estimator” in the Abstract, which is not correct.
4. Equation (2) exists if  is invertible.
5. To improve the literature and cover some existing results, the authors may refer to a couple of related studies such as Arashi et al. (2021a, 2021b), Norouzirad, and Arashi (2019), and Saleh et al. (2019). Since the core of this research is oriented on the multicollinearity problem and its defects in estimation, knowing remedies and strategies to combat multicollinearity comes to be important. The ridge regression (RR) estimation and other estimation strategies built upon it such as shrinkage ridge or scaled lasso thus are important strategies to be used in this context.
6. Provide more details for obtaining  in Eq. (11).
7. After Eq. (16), what is ?
8. For selecting ridge parameter  in Eqs (32)-(37), provide a reference.
9. In Eq. (37),  should read .
10. In Eq. (38), the pseudo normal variables are denoted by small ; however, capital  has defined the line after.
11. It is helpful to provide a table for the real data used in the Application, although a data availability statement is added.  

Is the work clearly and accurately presented and does it cite the current literature?

Partly

If applicable, is the statistical analysis and its interpretation appropriate?

Yes

Are all the source data underlying the results available to ensure full reproducibility?

Partly

Is the study design appropriate and is the work technically sound?

Yes

Are the conclusions drawn adequately supported by the results?

Yes

Are sufficient details of methods and analysis provided to allow replication by others?

Yes

Reviewer Expertise:

Shrinkage estimation; High-dimensional analysis; Penalized regression

I confirm that I have read this submission and believe that I have an appropriate level of expertise to confirm that it is of an acceptable scientific standard.

## References

[1] : SLASSO: a scaled LASSO for multicollinear situations. *Journal of Statistical Computation and Simulation* .2021; 10.1080/00949655.2021.1924174 1-14 10.1080/00949655.2021.1924174

[2] : Ridge regression and its applications in genetic studies. *PLoS One* .2021;16(4) : 10.1371/journal.pone.0245376 e0245376 10.1371/journal.pone.0245376 33831027PMC8031387

[3] : Preliminary test and Stein-type shrinkage ridge estimators in robust regression. *Statistical Papers* .2019;60(6) : 10.1007/s00362-017-0899-3 1849-1882 10.1007/s00362-017-0899-3

[4] : Theory of Ridge Regression Estimation with Applications. *Wiley* .2019;

# Peer review: Reviewer response for version 1

**DOI:** 10.5256/f1000research.58523.r92412

**Published:** 2021-09-13

**Authors:** Muhammad Amin

**Affiliations:** 1 Department of Statistics, University of Sargodha, Sargodha, Pakistan

**Report on manuscript “** **Unbiased K-L estimator for the linear regression model** **''**

 In this paper, the authors introduced a new unbiased KL estimator for the linear regression model to overcome the effect of multicollinearity. The paper is original and deals with a topic of interest. This paper can be indexed after incorporating the following points:

1. Page 1, paragraph 1, line 3, the estimator should be the OLS estimator
2. In the abstract, the conclusion is not appropriate, I request the authors to rewrite the conclusion
3. Page 3, above equation 2, "the model is defined as" should be "the OLS estimator is defined as"
4. Page 3, change “of the two-parameter estimator by Ozkale and Kaciranlar9.” to “of the two-parameter estimator based on the work of Ozkale and Kaciranlar9."
5. Page 3, introduction the last paragraph, change “a new unbiased technique is developed based on the K-L estimator” to “a new unbiased K-L estimator is developed for the linear regression model"
6. Below equation 4, "According to 16, the unbiased ridge estimator with the introduction", the authors should be clear is 16 an equation or a reference number?
7. Above equation 6,  “Modified ridge-type method proposed by 3 is given as follows”, here, indicate the author's name with reference number
8. Write the reason for your proposed estimator over other estimators in the last paragraph of the introduction section
9. The expression below equation 7, change 1 to I
10. Above equation 8, “Recently13, proposed the K-L estimator”, indicate here the author's names and reference numbers
11. In the results section line 1, change “The independent variables were generated” to “ The correlated explanatory variables were generated”
12. Equation 19 is not correct, authors should correct this equation
13. All simulation results behaviour is not indicated which should be indicated - when and which estimator over the other gives better performance?
14. There are some grammatical issues that should be corrected

Is the work clearly and accurately presented and does it cite the current literature?

Yes

If applicable, is the statistical analysis and its interpretation appropriate?

Partly

Are all the source data underlying the results available to ensure full reproducibility?

Yes

Is the study design appropriate and is the work technically sound?

Yes

Are the conclusions drawn adequately supported by the results?

Yes

Are sufficient details of methods and analysis provided to allow replication by others?

Yes

Reviewer Expertise:

Linear and generalized linear models, Biased Estimation Methods, Outlier Analysis

I confirm that I have read this submission and believe that I have an appropriate level of expertise to confirm that it is of an acceptable scientific standard, however I have significant reservations, as outlined above.

# Peer review: Reviewer response for version 1

**DOI:** 10.5256/f1000research.58523.r92414

**Published:** 2021-09-03

**Authors:** Oluwayemisi Oyeronke Alaba

**Affiliations:** 1 Department of Statistics, University of Ibadan, Ibadan, Nigeria

In the abstract, inefficiency is better used than performance drops when multicollinearity is present. The new unbiased estimator should be clearly stated and the reason why there is a need to modify it, which was clearly stated in the body of the work.

 Why did you choose the biasing parameter - did you check the unbiasedness of KL estimator, what were the limitations in previous studies before the need to modify it? You only stated that you classified it as a biased estimator with a single biasing parameter. The results of the simulation did not display well, but it will be better if few figures could be picked to discuss the results

Is the work clearly and accurately presented and does it cite the current literature?

Yes

If applicable, is the statistical analysis and its interpretation appropriate?

Yes

Are all the source data underlying the results available to ensure full reproducibility?

Yes

Is the study design appropriate and is the work technically sound?

Yes

Are the conclusions drawn adequately supported by the results?

Yes

Are sufficient details of methods and analysis provided to allow replication by others?

Yes

Reviewer Expertise:

Statistical Modelling, Econometrics

I confirm that I have read this submission and believe that I have an appropriate level of expertise to confirm that it is of an acceptable scientific standard.
